# Multilingual media project as a tool for reducing stigma in psychiatry: the experience of the Tomsk National Research Medical Center

**DOI:** 10.1192/j.eurpsy.2026.10993

**Published:** 2026-07-31

**Authors:** N. A. Bokhan, V. Lebedeva, E. Gutkevich, S. Vladimirova

**Affiliations:** Mental Health Research Institute, Tomsk, Russian Federation

## Abstract

**Introduction:**

Stigma remains a key barrier to mental health care. Digitalization creates new opportunities for education and support. This paper presents the results of a multilingual media project aimed at overcoming stigma and creating an inclusive environment.

**Objectives:**

To develop and implement a comprehensive information and communication platform to support patients with mental disorders and their families, reduce stigma, and integrate Russian research into the global scientific community.

**Methods:**

A media project, “Creating Connections and Helping,” was initiated. It includes an official website, a scientific journal in Russian and English, and a series of specialized blogs. The scientific basis was confirmed by a 15-year screening survey (n = ~5,000) to assess the need for such resources. Online platform traffic metrics over a two-year period were used to analyze effectiveness.

**Results:**

98% of respondents confirmed a high demand for specialized online resources. Traffic analysis revealed sustained interest: the project’s blogs garnered approximately 5,000 views. Content combining personal experience and practical recommendations proved to be the most popular. The project facilitated international collaboration with scientists from Canada and Italy and contributed to the indexing of the Tomsk National Research Medical Center journal in the Scopus database.

**Discussion:**

The results demonstrate the high social demand for digital platforms that offer not only information but also emotional support. The project’s success is due to its practical focus and multilingual format, which helps overcome cultural and language barriers. Expert review highlighted areas for improvement, including in-depth statistical analysis and stronger links to national health goals.

**Conclusions:**

The Tomsk National Research Medical Center’s multilingual media project has proven effective as a practical tool for reducing stigma and supporting the community. This model can be adapted in other countries to promote mental health based on principles of inclusiveness and scientific knowledge.

**Disclosure of Interest:**

None Declared

